# Characteristics

**Published:** 1895-03

**Authors:** F. E. Battershell

**Affiliations:** New Philadelphia, Ohio


### Characteristics.

Read before the Ohio State Dental Society, December, 1894.

BY F. E. BATTERSHELL, D.D.S., NEW PHILADELPHIA, OHIO.

It is commonly believed that the brute deprived of the soul accompanied by power of reason, is endowed with the subtle faculty of instinct, as a recompense. This mental counterpoise of instinct for reason, according to the superficial observer, must needs give to the soulless and subjected animal the entire stock of instinct to render the life balance even. By this its own sensate faculty, it is enabled to grasp instantly and automatically the various circumstances of its environment for advantage in its existence. The fact that man is blessed with the more excellent gift, seems to overshadow the possibility—the truth—that the nobler creature is furnished with both elements of intellect, either of which may serve, as occasion calls for the better.

Assuming, therefore, that the inferior creature is no monopolist in the singular quality—instinct,—it may be allowed, that, in sharing, the greater degree is retained because of the greater need.

Man's reason towers so above the faculty of instinct that he is oblivious of its influence upon his actions. Oft what he attributes to his own volition, is but the co-ordination of those sets of reflexes, by which certain movements correspond to particular irritants, through vision, smell, hearing, and the like.

These correspondences are wrought by established associations. We see an ambling biped enter our front gate. His visage is unkempt and his wilted garments droop upon his person. We do not stop to question, or enter upon a process of reasoning, but jump at the conclusion that the object focused on our retina is a tramp. Our family dog by his instantaneous process, jumps at the same conclusion with results more effective and much more disastrous to the aforesaid biped.

Habit and instinct are near akin. What one has done many times is done with thoughtless perfection. The garrulous female unmindful of " the base degrees by which she did ascend," becomes a Mother Grundy. How the uncouth plebeian becomes the honored patrician, is as much a consequence of habit betrayed, as of successful adventure in politics. "Thou hast been faithful over a few things, I will make thee ruler over many," is more often the *fiat* of the commonweal, than political bosses admit, or the fortunate themselves suspect.

If the savage animal recognizes in the *genus homo* his foe and master, how much more do the kindred, that are qualified with reason *and* with intuition, discern the variations in *their* kinds? That the occult faculty has more to do in discriminating conditions among men than is ordinarily recognized, we presume is now brought within view. Seeing, then, the consort of reason, the swift interpretor of our ways, is instinct. Therefore let us make our professional call and election sure, by rendering the peculiar homage the dame requires for recognition.

The ecclesiastic is known by his cloth and long face; the family doctor by his pre-occupied air, hurried step, and apothecary odor; the merchant by his alert and accommodating address, mercenary smile, and a pencil stuck aslant his ear; the soldier by his commanding carriage and clock-like movements; the lawyer by the midnight air of mystery in his eye, the ultra respectability of his features, and the great depths to which his hands and thoughts seem to descend, betimes, into his trousers pockets; and the dentist by his dyspeptic countenance and the charm of his watch-chain: "Hold!" cries the lawyer, "I object. These outward circumstances are not competent evidence in a court of equity. They oft mislead. There is an indefinable sense within the average mind which enables it to identify the occupation of the person separated from his work and away from the field of labor. Like some strange dream which startles the wakeful senses by the accuracy of the circumstances, past or near approaching; or like a fit of abstraction, when the mental activities are not co-ordinated with the physical reflexes, and an absurd act is committed by the individual, because the faculties cast out of this "star chamber" council of the mind, have acted independently according to habit, but out of correspondence with the superior court. So the nimble mind, with formulas acquired from habit, is constantly engaged with tests, and is even submitting reports for the guidance of the understanding."

That this process, translated intuition, produces a correct formula for the identification of a dentist, may be discovered by learning what intelligent mind owners count the essential ingredients for the dental compound. The equation should evenly balance, opinion for merit, reputation for achievements. If the formula is imperfect, it is because the ideals, *i. e.* typical dentists, are insufficiently numerous, and a prevailing accurate impression is yet uncreated.

There are counterfeits and imitations; as well of worthy men, as of money and merchandise. A gentlemanly bearing more than a miniature set of false teeth, or a naked three pronged molar, pendulating from the vest pocket; a cheerful, assuring voice more than an exhibition of much gold in the front teeth and a readiness to explain its presence; quiet culture and trim keeping rather than extravagance, lead to a correct conclusion in an inquiry. A warm soul in a healthy body is a good token, but not a sure sign; for, like "the shadow of a rock in a weary land," it may not always be present. The horny hand of the farmer, the strong hand of the laborer, the mobile hand of the mechanic, the deft and careful hand of a dentist, unlike the untrained hands of men in other professions, soft and blue veined though they be.

A fair countenance will abridge the test. Abstainence from tea and coffee will clear the complexion, steady the nerves, and brighten the eye.

Tobacco, like alcohol, is an incompatible; either one would precipitate any good solution, or give it in as a vulgar associate of "men only." The doe takes the scent of the stealthy hunter, yet out of sight, and is away; so the gentle lady sniffs the vile smell of tobacco, or an intoxicant, and with the hauteur of offense, stops not to consider.

There is that ever nearness or presence of pain, that close observance of its results, that control of the subject and of self, which must beget one of the elements of this formula—sympathy. There is that knowledge of peculiar facts regarding the substitutes and realities of the patient's mouth, eccentricities of disposition, and delicate experiences developed by necessarily close relations of the chair which will supply another quality, the most needful and patent in the perscription; it may well be defined the antiseptic, viz: circumspection. Without this ingredient no compound is genuine, it can not be kept pure, will not exhibit the true color, and is liable to effervesce and lose strength after the removal of the stopper.

In some compositions there is found a small proportion—a few minims perhaps—of a bitter tincture—hypersensitiveness—which, though of little consequence in the general effect, renders the particular mixture somewhat disagreeable. The person bearing this aptitude gags at the familiar "doc" or "mister" unmindful that friends often substitute or abbreviate titles to lengthen their affection or respect for those whom they wish to honor most. Is it a *haute nouveaute* of form. This title of Doctor? If it be uttered in a calling voice, on the thoroughfare of any considerable town, a goodly dozen of heads may turn to reply—M.D.'s, D.D.'s, Ph. D.'s, V.S.'s, LL.D.'s, and possibly some Doctors of Dental Surgery. The title letters of the renowned Agassiz were L. J. R., these preceded his cognomen; the word "teacher" followed to define his vocation. Thus his name, in full, ran Lewis John Randolph Agassiz, teacher. Even this, his own modest choice of name, is abridged by a worshipful and affectionate world. "Agassiz," alone, stands out in bold relief over all titles and acquirements of honor. "What's in a name? that which we call a rose, by any other name would smell as sweet."

Let us therefore fill the bottle. Pour in patience, carefulness, cheerfulness, temperance, skill, invention, conscience, culture, prudence. Fill it until it overflows—it will stretch and widen—keep filling; the overflow is what the world gets, the outflow is our garment of beauty or our covering of rags, according to the contents. What is within is ever circulating outward. A shallow vessel is soon exhausted by evaporation. From a deep vessel the overflow is more abundant, is cool, is quiet, and is exhausted last. Do not pump in from beneath; pour in from above—from books, experience, words of the elders. The good men and superiors in the profession, and from the higher inspiration which descends upon every one who is not slothful in business.

